# Transcriptome characterisation and simple sequence repeat marker discovery in the seagrass *Posidonia oceanica*

**DOI:** 10.1038/sdata.2016.115

**Published:** 2016-12-20

**Authors:** D. D’Esposito, L. Orrù, E. Dattolo, L. Bernardo, A. Lamontara, L. Orsini, I.A Serra, S. Mazzuca, G. Procaccini

**Affiliations:** 1 Stazione Zoologica Anton Dohrn, Villa Comunale, 80121 Napoli, Italy; 2 Consiglio per la ricerca in agricoltura e l’analisi dell’economia agraria, Centro di ricerca per la genomica vegetale, 29017 Fiorenzuola d’Arda, Italy; 3 Laboratorio di Biologia e Proteomica Vegetale (Lab. Bio. Pro. Ve.), Dipartimento di Chimica e Tecnologie Chimiche, Università della Calabria, 87036 Rende (CS), Italy; 4 Environmental Genomics Group, School of Biosciences, University of Birmingham, Birmingham, B15 2TT, UK

**Keywords:** Plant sciences, Computational biology and bioinformatics

## Abstract

*Posidonia oceanic*a is an endemic seagrass in the Mediterranean Sea, where it provides important ecosystem services and sustains a rich and diverse ecosystem. *P. oceanica* meadows extend from the surface to 40 meters depth. With the aim of boosting research in this iconic species, we generated a comprehensive RNA-Seq data set for *P. oceanica* by sequencing specimens collected at two depths and two times during the day. With this approach we attempted to capture the transcriptional diversity associated with change in light and other depth-related environmental factors. Using this extensive data set we generated gene predictions and identified an extensive catalogue of potential Simple Sequence Repeats (SSR) markers. The data generated here will open new avenues for the analysis of population genetic features and functional variation in *P. oceanica*. In total, 79,235 contigs were obtained by the assembly of 70,453,120 paired end reads. 43,711 contigs were successfully annotated. A total of 17,436 SSR were identified within 13,912 contigs.

## Background & Summary

*Posidonia oceanic*a is an endemic seagrass species in the Mediterranean Sea where it forms extensive meadows along the coastline, from the surface to 40 m depth^1^. *P. oceanica* provides important ecosystem services including sediment stabilization, contribution to CO_2_ absorption, sheltering and food sources for a diverse associated community, which also includes commercially important fish species^2^. The total mapped extent of *P. oceanica* is estimated about 1.2E+6 ha^3^ with an estimated economic value of 1.7E+6 € ha y^−1^ (ref. 2). *P. oceanica* meadows are declining throughout the Mediterranean basin, due to direct and indirect effect of human activities^3^, with important consequences on the stability of the coastline and on the whole set of ecosystem services provided by meadows. Plants can respond to changes adopting meadow-scale adaptive strategies or can exhibit organismal-scale responses, adjusting fitness and performance. Understanding how human-driven environmental changes impact marine plants and how plants respond to changes is critical to design appropriate conservation strategies. However, our understanding of the consequences of the human impact on *P. oceanica* meadows is hampered by the absence of specific molecular tools. To date, a quite extensive literature exists on the genetic diversity and structure of the species, using a limited set of SSR (Simple Sequence Repeats) markers e.g., refs 4–6. Nevertheless, the limited number of markers and the neutral behavior of most of them, did not allow to detect clear signature of selection along environmental and geographic gradients. Only a limited number of functional studies has been conducted on *P. oceanica*, mostly looking at the expression of selected target genes e.g., refs 7–10. A first set of annotated genes was developed for *P. oceanica*, reconstructed from samples collected at different depths^10^, and a SSH (Suppression Subtractive Hybridization) library was built in order to detect differentially expressed genes^7,8,11^. These resources constitute an important first step towards the characterization of the transcriptome of the species, but the limited number of sequences is not at all representative of the whole transcriptome of the species. Here we make a step forward, building a complete transcriptome that can be interrogated to investigate the effect of human-driven environmental change on the endemic seagrass species *P. oceanica*, also taking advantage of the recent release of the first seagrass genome from the temperate species *Zostera marina*^12^.

*P. oceanica* is a clonal grass species, with lignified rhizomes and a bundle of erect strip-like leaves. Sexual reproduction is not regular and the flower is hermaphroditic. Shallow and deep portions of the same meadow are usually genetically distinct (e.g., ref. 13). We generated a *de novo* transcriptome assembly by sequencing specimens collected at two different depths and at different times of the day to capture representative transcriptional changes associated with photoperiod and habitat distribution in our species. The assembled contigs were annotated, generating the first gene catalogues for *P. oceanica*. In addition we interrogate the newly assembled transcriptome to identify Simple Sequence Repeats. Next generation sequencing (NGS) technologies recently become the methods of choice for gene expression analysis due to their unprecedented level of sensitivity and high-throughput nature^14^. RNA-seq has also been shown to be a very efficient, cost-effective approach for transcriptome analysis of non-model species. In fact, also in absence of the genome, RNA-seq allows construction of complete transcriptome of an organism by *de novo* assembly^15^.

The catalogue of annotated *P. oceanica* genes together with the SSR provides the seagrass community with advanced tools to link the effect of human-induced environmental change and functional responses in *P. oceanica*, and molecular markers useful in population genetic studies. These resources will be of critical importance to implement the conservation strategies aimed at preserving this iconic species.

## Methods

### Sampling design and RNA extraction

**Figure 1:**
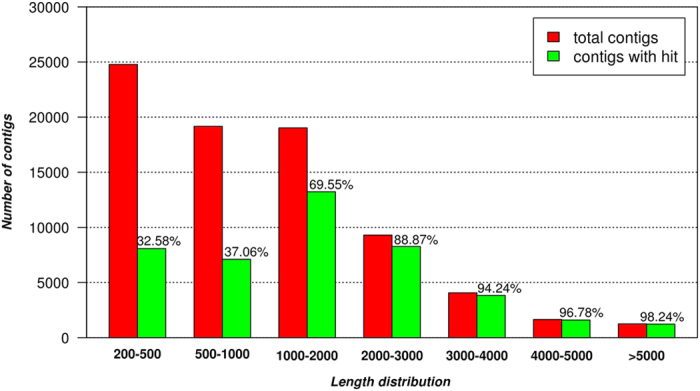
Effect of contigs length on the percentage of BLAST hits against NR protein database. The red bars indicate the length distribution of the contigs, the green bars indicate the number of contigs with a blast hit.

*Posidonia oceanica* shoots were collected in 2012 from a meadow located in Stareso (Corse, 8°45′E, 42°35’N, Fig. 1). Sampling was performed at two different depths, −5 m (shallow station) and −25 m (deep station) and at two different times of the day (day and night). Sampling was performed in the framework of a comprehensive study aimed to characterize *P. oceanica* meadows, from the plant to the whole ecosystem^16^, in collaboration with international partners within the EU COST Action ES-0906 (Seagrass productivity: from genes to ecosystem management). Leaf tissue from an adult shoot from each depth and day-time was cleaned from epiphytes, stored in RNA later solution (Life Technologies) overnight at 4 °C and then transferred at −20 °C. Total RNA was isolated using the Aurum Total RNA mini kit (Bio-Rad) following the manufacturer’s instructions. The quality of total RNA was checked using NanoDrop technologies (ND-1000 Spectrophotometer, Peqlab) and the Agilent 2100 Bioanalyzer (RNA Nano Chip, Agilent)^17^.

### Transcriptome sequencing, assembly and annotation

Libraries were constructed starting from 3.5 μg of total RNA using the TruSeq RNA Sample Preparation Kit. Paired ends libraries (95 bp×2) were sequenced on an Illumina Genome Analyzer IIx instrument (Illumina, San Diego, California, USA) following manufacturer instructions. Prior to assembly, raw reads were filtered with Prinseq v0.20.4 (ref. 17), in order to remove duplicated sequences and the reads showing a quality score mean below 30 (Q<30) and with Cutadapt v1.9, to remove adapters (Table 1). High quality reads were pooled and used for the *de novo* assembly using Trinity v2.1.1 (ref. 18). Trinity was run using the following parameters: the fixed default k-mer size of 25 and a minimum contig length of 200 bp. Botwie v1.1.2 (ref. 19) was utilized to align the reads to the contigs and BEDTools 2.25.0 (ref. 20) was used to calculate the coverage depth. Contigs were cleaned from redundant sequences using CD-HIT program v4.6 (ref. 21). To assess the completeness of the transcriptome assembly, we used the 458 Core Eukaryotic genes (KOGs) obtained from CEGMA (http://korflab.ucdavis.edu/datasets/cegma/) to measure how the (KOGs) set is covered by the *P. oceanica* transcriptome. Analysis was performed using the TransRate software^22^ which makes use of the reciprocal best blast hit methods to compare a transcriptome against a reference. The obtained contigs were annotated by blast search (BLASTx) against the NCBI nr protein database or by conducting a local BLASTx search. The databases queried with a local BLASTx search included the *Vitis vinifera* proteins, *Phoenix dactylifera* proteins and *Elaeis guineensis* proteins, downloaded from NCBI GenBank, *Zostera marina* proteins downloaded from ORCAE^23^ Clusters of Orthologous Group (COG)^24^. Information about domain/motifs content of the sequences were retrieved using the InterProScan functionality in Blast2GO v3.1.3 (ref. 25). To classify the function of contigs, GO assignment was performed using Blast2GO software.

### Detection of SSR containing sequences.

SSR were identified using the MISA MicroSatellite identification tool (http://pgrc.ipk-gatersleben.de/misa/), defining a minimum repeat length criteria of six repeated units for dinucleotides, five repeated units for tri, tetra, penta and hexanucleotides. We also identified compound SSR as the sequences in which two SSR were separated by no more than 10 bases.

## Data Records

The project was submitted to NCBI BioProject with BioProject ID: PRJNA315106 (Data Citation 1). The assembled contigs were deposited at DDBJ/ENA/GenBank under the accession GEMD01000000 (Data Citation 2).

## Technical Validation

In order to characterize and comprehensively cover the transcriptome of *Posidonia oceanica*, RNA was isolated from plants living at different depth (shallow and deep) and in different times of the day (day and night). Four libraries were prepared and sequenced using the Illumina sequencing technology. A total of 86,691,134 reads were generated. After filtering, 70,453,120 high quality reads were obtained and used for *de novo* assembly (Table 1). Reads were assembled using the Trinity software obtaining a total of 165,235 contigs. The reads used to create the assembly were mapped back to the assembly using Botwie and the coverage depth calculated. Contigs showing a coverage less than a threshold of 5X were removed. CD-HIT clustering program was used to remove sequence redundancy by applying a global identity threshold of 90% and retaining the longest possible contigs. After these filtering steps, 79,235 contigs remained. As shown in Table 2, the contigs lengths ranged from 201 to 16,705 bp, with an average length of 1,278 bp. The contigs size distribution is shown in Fig. 1. The TransRate software was used to test the completeness of the transcriptomic assembly by assessing the coverage of the CEGMA set of 458 KOGs. TransRate aligns each contig of the assembly with the conserved set of KOGs using the Conditional Reciprocal Best Blast CRBB (http://hibberdlab.com/transrate/metrics.html#the-contig-score). A total of 340 KOGs showed a coverage of 95% while 418 KOGs had a 85% coverage (Table 3). These results suggest that the assembly is a reliable representation of the *P. oceanica* transcriptome.

**Figure 2:**
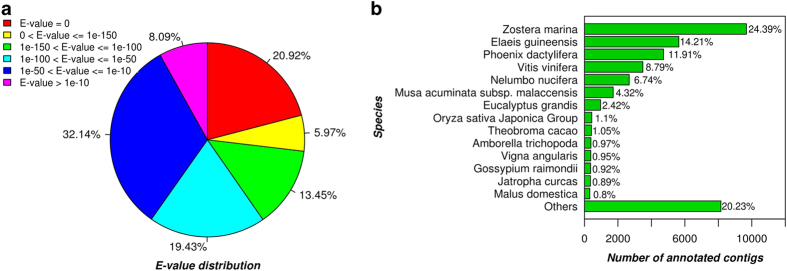
Characteristics of homology search of contigs against the NR protein database. (**a**) E-value distribution of the top BLAST hits for each contig (E-value ≤1.0e-5). (**b**) Hit species distribution.

Functional annotation of the *P. oceanica* transcriptome was based on two levels of sequence similarity namely sequence-based and domain-based alignments^26^. Sequence based alignments were performed by blast search (BLASTx) against the non-redundant protein database (NR) in NCBI and by local BLASTx against the proteins of the four top hit species identified from the BLASTX against the NR protein database. In Fig. 1 the effect of the sequence length on the percentage of significant matches in BLASTx results is reported. The data shown in Fig. 1 make evident that the longer the length of the assembled sequences the greater the number of matches. The E-value distribution in Fig. 2a revealed that the 59.7% of the contigs with hit have a strong homology with the sequences available in the NR protein database (E value<1.0e-50) while the remaining 40.2% show a match with an E-value above 1.0e-50. In Fig. 2b, the BLASTx top hit species distribution of matches with known sequences is reported and indicate that the majority of *P. oceanica* contigs show the highest homology with *Z. marina* sequences (24.39%). The others most represented species included *E. guineensis* (14.21%), *P. dactylifera* (11.91%) and *V. vinifera* (8.79%). All the alignments have been carried out setting the E-value thresholds at the value of ≤1e-5.

**Figure 3:**
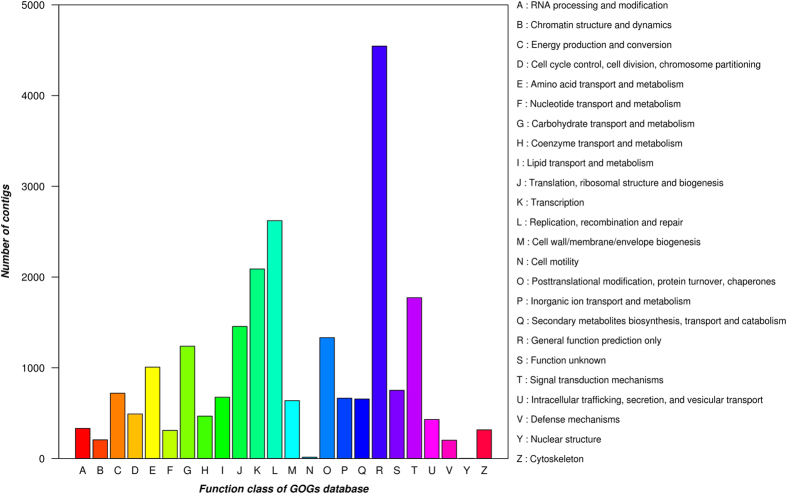
COG functional classification of the *Posidonia oceanica* transcriptome.

Annotation was completed aligning the contigs against COGs and using the functionality of InterProScan in Blast2GO software. 22,035 assembled contigs matched entries against the InterProScan queried protein databases (Table 4). A local BLASTx was used to align the assembled contigs against the COGs database^21^ to predict and classify possible function. 17,421 COG functional annotations were obtained that were grouped into 24 functional categories (Fig. 3). The annotation results are summarized in Supplementary Table S1.

**Figure 4:**
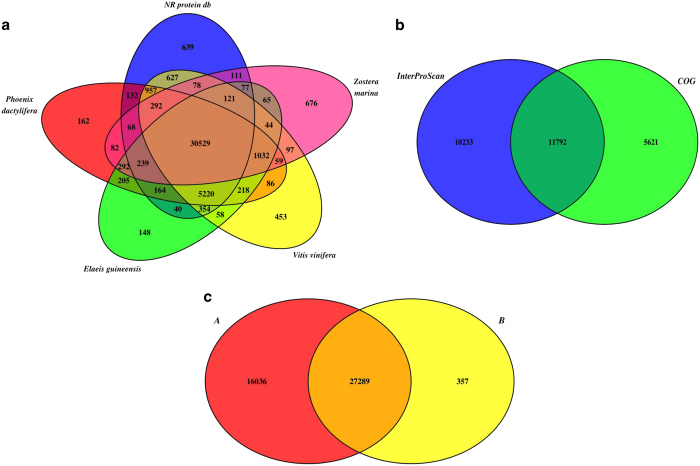
Venn diagram showing distribution of similarity search results. (**a**) BLAST results against NCBI NR protein database, *Zostera marina* proteins, *Elaeis guineensis* proteins, *Phoenix dactylifera* proteins and *Vitis vinifera* proteins. (**b**) Annotation from InterPro and COGs. (**c**) Summary of the annotation obtained in **a** and **b**.

**Figure 5:**
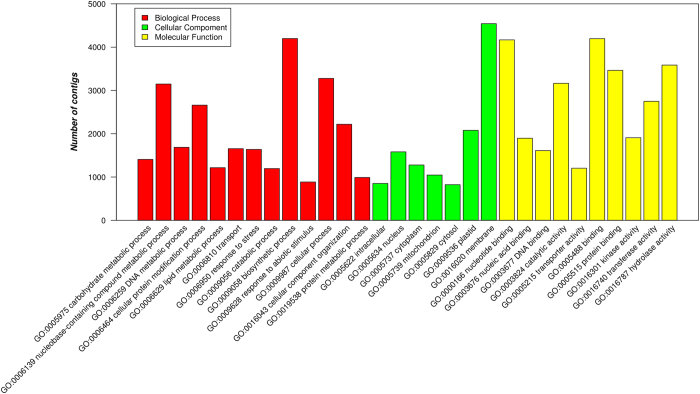
Histogram presentation of Gene Ontology classification. The results are summarized in three main categories: biological process, cellular component and molecular function. The right and left y-axis indicates the number and the percentage of contigs in a sub-category, respectively.

The Fig. 4 shows Venn diagrams illustrating the distribution of similarity search results. In the Venn diagram in Fig. 4a the BLAST results against NR protein, *Z. marina* proteins, *E. guineensis* proteins, *P. dactylifera* proteins and *V. vinifera* proteins are reported. The total number of annotations obtained from these databases was 43,325 (54.6% of total final contigs). In Fig. 4b, the annotation results obtained from COGs and InterProScan are shown. The number of contigs which show a hit against these databases was 27,646 (34.8% of total final contigs). In the Venn diagram in Fig. 4c, all the annotation resulting from the databases in Fig. 4a,b are reported. Integrating the results obtained from all the databases queried, a total of 43,682 sequences (55% of assembled contigs) resulted annotated. Blast2GO PRO was used to assign Gene Ontology (GO) term and functionally categorize the assembled *P. oceanica* contigs. At least one GO is assigned to 25,495 assembled contigs (32.1% of total contigs; Fig. 5).

The annotated transcript sequences here reported represent a significant improvement of the genomic information available for this species.

The 79,235 contigs obtained from the assembly were mined for the identification of contigs containing SSR. The search criteria adopted allowed the identification of 17,436 SSR contained in 13,912 contigs (Supplementary Table S2 and Table 5). The most abundant repeat type was trinucleotides (58%) and dinucleotides (38%). The most abundant repeat motif was AG among dinucleotides (18% of the dinucleotides repeats), GGA among trinucleotides (6.4% of the trinucleotides repeats), AAAG among tetranucleotides (8.3% of the tetranucleotides repeats). Once the SSR here discovered will be tested for polymorphism, this will increase the SSR markers resources for this species and will be of help in population genetics studies. Furthermore, because these SSR were found in the transcribed part of the genome they will greatly help the studies aimed to identify the genes involved in the adaption of *P. oceanica* in the deep-sea environment.

## Additional information

**How to cite this article**: D’Esposito, D. *et al.* Transcriptome characterisation and simple sequence repeat marker discovery in the seagrass *Posidonia oceanica*. *Sci. Data* 3:160115 doi: 10.1038/sdata.2016.115 (2016).

**Publisher’s note**: Springer Nature remains neutral with regard to jurisdictional claims in published maps and institutional affiliations.

## Supplementary Material

Supplementary Table S1

Supplementary Table S2

## Figures and Tables

**Table 1:** Summary of data before and after raw reads filtering.

**Sample**	**Raw reads**	**Cleaned reads**	**Cleaned reads %**
DE3	22,325,705	18,111,963	81.13
F2-2	22,780,790	18,400,802	80.77
E1	20,698,407	16,781,903	81.08
F1	20,886,232	17,158,452	82.15
Total	86,691,134	70,453,120	81.27

**Table 2:** Summary of *Posidonia oceanica* contigs.

	**N° contig**	**N° bases (Mbp)**	**N50 (bp)**	**Shortest (bp)**	**Longest (bp)**	**Mean (bp)**
Contigs	79,235	101.304	2,041	201	16,705	1,278.53

**Table 3:** Results obtained comparing the *Posidonia oceanica* transcriptome assembly with the 458 KOGs with TransRate. RBB hits: reciprocal best blast hit.

**Analysis**	**Results**
Contigs with RBB hit	919
% Contigs with RBB hit	1%
KOGs with RBB hit	457
% KOGs with CRBB hit	100%
KOGs with 25% coverage	457
% KOGs with 25% coverage	100%
KOGs with 50% coverage	457
% KOGs with 50% coverage	100%
KOGs with 75% coverage	440
% KOGs with 75% coverage	96%
KOGs with 85% coverage	418
% KOGs with 85% coverage	91%
KOGs with 95% coverage	340
% KOGs with 95% coverage	74%

**Table 4:** Summary of annotation against the domain/family databases queried with InterProScan.

**DATABASE**	**Number of contigs with a match**	**contigs %**	**Protein domain/family**
Gene3D	13,239	16.7	1,032
Hamap	305	0.38	195
InterPro	20,244	25.55	5,772
PANTHER	21,867	27.6	12,129
Pfam	18,064	22.8	3,502
PIRSF	670	0.85	356
PRINTS	2,225	2.81	397
PRINTS	212	0.27	86
ProSitePatterns	3,264	4.12	588
ProSiteProfiles	7,504	9.47	550
SMART	5,177	6.53	507
SUPERFAMILY	13,933	17.58	907
TIGRFAM	2,018	2.55	608

**Table 5:** Summary of SSR detected from *Posidonia oceanica* contigs.

**Parameters**	**Total number**	**Percentage**
Number of contigs containing SSRs	13,912	17.5
Number of SSRs identified from *Posidonia* transcript sequences	17,436	
Contigs containing more than 1 SSR	3,369	24.2
Number of SSR present in compound formation	869	4.7
Dinucleotides repeat units	7,012	38.2
Trinucleotides repeat units	10,602	57.9
Tetranucleotides repeat units	465	2.5
Pentanucleotides repeat units	96	0.5
Hexanucleotides repeat units	130	0.7
