# Corrigendum: Transcriptome characterisation and simple sequence repeat marker discovery in the seagrass *Posidonia oceanica*

**DOI:** 10.1038/sdata.2017.25

**Published:** 2017-03-14

**Authors:** D. D’Esposito, L. Orrù, E. Dattolo, L. Bernardo, A. Lamontanara, L. Orsini, I.A Serra, S. Mazzuca, G. Procaccini

**Keywords:** Plant sciences, Computational biology and bioinformatics

*Scientific Data* 3:160115 doi: 10.1038/sdata.2016.115 (2016); Published 20 December 2016; Updated 14 March 2017

The original version of this Data Descriptor contained a typographical error in the spelling of the author A. Lamontanara, which was incorrectly given as A. Lamontara. This has now been corrected in the PDF and HTML versions of the Data Descriptor.

